# ICTV Virus Taxonomy Profile: Apasviridae 2026

**DOI:** 10.1099/jgv.0.002230

**Published:** 2026-03-18

**Authors:** Apoorva Prabhu, Christian Rinke

**Affiliations:** 1 School of Chemistry and Molecular Biosciences, Australian Centre for Ecogenomics, The University of Queensland, St Lucia, QLD 4072, Australia; 2 Sydney Institute of Marine Science, New South Wales 2088, Mosman, Australia; 3 Climate Change Cluster (C3), University of Technology Sydney, Ultimo, NSW 2007, Australia; 4 Department of Microbiology, University of Innsbruck, 6020 Innsbruck, Austria

**Keywords:** *Apasviridae*, ICTV Report, magroviruses, marine archaea, *Poseidoniales*, taxonomy

## Abstract

The family *Apasviridae* includes dsDNA viruses associated with the marine archaeal lineage *Poseidoniales*. Members of this family have been identified using metagenomic analyses of brackish estuarine samples and are related to other ‘magroviruses’ infecting *Poseidoniales* archaea. The family belongs to the order *Magrovirales* and includes the genus *Agnivirus* and the species *Agnivirus brisbanense*. Viruses in the family possess a linear dsDNA genome of about 108 kbp and encode modules for DNA replication and virion morphogenesis, such as those relating to the formation of an icosahedral capsid and a helical tail, characteristic of members of the class *Caudoviricetes*. This is a summary of the International Committee on Taxonomy of Viruses (ICTV) Report on the family *Apasviridae*, which is available at ictv.global/report/apasviridae.

## Virion

Members of the order *Magrovirales* (magroviruses) have so far been discovered exclusively through metagenomic surveys [1,5] and comprise several recognized families. Within this group, the family *Apasviridae* corresponds to clade A of the order *Magrovirales*. The complete genome of magrovirus_A_01 (species *Agnivirus brisbanense*) encodes a morphogenetic module, including proteins for head and tail formation, such as the major capsid protein, the portal protein and the tail fibre protein, which are typical of viruses in the class *Caudoviricete*s (Table 1). In addition to these proteins, the close relationship with tailed haloviruses (family *Druskaviridae*) suggests that apasvirids likely possess icosahedral heads and helical tails [6,7].

**Table 1.** Characteristics of members of the family *Apasviridae*

Example	magrovirus_A_01 (OR863078), species *Agnivirus brisbanense*
Virion	Predicted icosahedral capsid and helical tail
Genome	Linear dsDNA of 108 kbp
Replication	Predicted virus-encoded DNA replisome, including a DNA polymerase family B
Translation	Unknown
Host range	*Poseidoniales* (Archaea)
Taxonomy	Realm *Duplodnaviria*, kingdom *Heunggongvirae*, phylum *Uroviricota*, class *Caudoviricetes*, order *Magrovirales*; includes the genus *Agnivirus* and the species *Agnivirus brisbanense*

## Genome

**Fig. 1.**
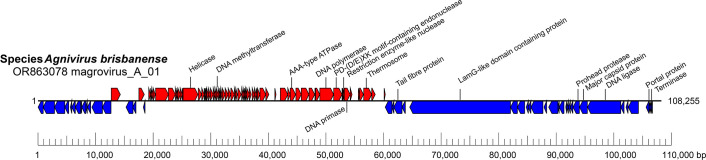
Genome organization of magrovirus_A_01. Boxes indicate open reading frames, as annotated on GenBank accession OR863078. Selected encoded products are labelled.

Members of the order *Magrovirales* are dsDNA viruses with a genome of 90–110 kbp, these being among the largest genomes described for archaeal viruses. The complete genome for magrovirus_A_01 is 108,255 bp, was assembled as a circular contig and is predicted to encode 99 proteins [5]. The genes comprising the morphogenetic module and the DNA replication and repair module are predicted to be transcribed in opposite directions (Fig. 1).

## Replication

Apasvirid genomes do not encode an integrase, and so are predicted to lead a lytic lifestyle. Apasvirids encode a set of proteins involved in genome replication and repair, including DNA polymerase family B (polB), archaeal-like DNA primase, PD-(D/E)XK motif-containing endonuclease, restriction enzyme-like nucleases, helicase, ATP-dependent DNA ligase and other proteins [5], suggesting that apasvirids do not rely on, or only partly rely on, the host replication machinery.

## Taxonomy

**Fig. 2.**
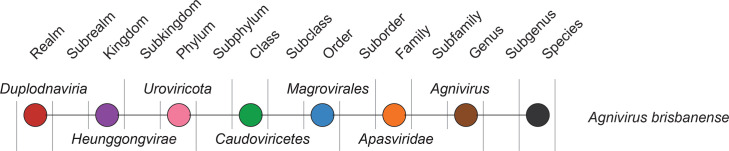
Relationships of taxa related to the family *Apasviridae*.

Current taxonomy: ictv.global/taxonomy. Members of the family *Apasviridae* are closely related to other members of the order *Magrovirales*, viruses which are associated with the marine archaeal order *Poseidoniales* (formerly known as Marine Group II Archaea) [3]. The order *Magrovirales* is composed of clades A (*Apasviridae*), B (*Aoguangviridae*) [8], C, D, E (*Krittikaviridae*) [9] and X. *Apasviridae* (pronounced Ap-as), named after the ancient water goddesses Apas in Indian mythology, includes the genus *Agnivirus* (pronounced Ag-nee) and the species *Agnivirus brisbanense* (Fig. 2), named after the Hindu God Agni and the isolation source, the Brisbane River. The family may include additional genera [5], but the complete genomes for these viruses are currently lacking, precluding their formal classification.

## Resources

Full ICTV Report on the family *Apasviridae*: ictv.global/report/apasviridae.

## Abbreviations

ICTV: International Committee on Taxonomy of Viruses
